# Supplementary material for: Factors Associated with Fatigued Driving among Australian Truck Drivers: A Cross-Sectional Study
Source: Int J Environ Res Public Health. 2023 Feb 3;20(3):2732. doi: 10.3390/ijerph20032732 (PMC9916394; doi:10.3390/ijerph20032732)
Supplement: Supplementary file 1 [file ijerph-20-02732-s001.zip › ijerph-2193626-Supplementary.pdf]

Supplementary Material

Table S1 Measurements of each determinant and collapsed categories

Factors	Determinants	Measurement	Example questions	Collapsed categories
Personal factors	Family situation	Questions regarding partnership status and dependent children	Which of the following best describes you?<br>How many dependent children do you have?	-No partner, no children<br>-No partner with children<br>-Partnered with no children<br>-Partnered with children
	Education	Highest level of education	What is the highest level of education and training you have completed?	-High school or lower<br>-Above high school
	Financial stress	Capacity to raise $2000 within 2 days as individual or household (scale of 1 to 10 where 1 is not at all stressed and 10 is as stressed can be)[41]	What do you feel is the level of your financial stress today?	-High (score of 6 to 10)<br>-Low (score of 1 to 5)
Occupational factors	Work task*	9 items adapted from an Occupational Health & Safety (OHS) vulnerability measurement using a 5-point Likert scale (No risk=Neve, Less than once a month or monthly; Yes to risk=Weekly or Daily/Almost daily)	In the last 12 months, how often in your job did you manually lift, carry or push items heavier than 20 kg at least 10 times during the day?	Low-risk group (1-4 risk items)<br>High-risk group (5-9 risk items)
	Time and schedule*	Five questions designed to measure scheduling, timing and delays	In the last 12 months, in your experience, how often did you arrive on time but were forced to wait to enter a dock?	-Low-risk group (1-2 risk items)<br>-High-risk group (3-5 risk items)
	Paid for delays*	One question designed to payment for delays	Thinking about the last 12 months, in your experience, you were paid for delays or waiting time.	Binary (No/Yes)
Work environment factors	Work environment*	7 items adapted from the OHS Vulnerability Scale	In the last 12 months, how often in your job did you experience discomfort by mechanical vibration or shock in your work?	-Low risk group (1-4 risk)<br>-High-risk group (5-7 risk)
	Workplace violence	Four items about drivers' experience of workplace violence	In the last 12 months, have you been verbally abused in workplace?	Binary (No/Yes)

Regulation factors	OHS training	9 types of OHS training		- “Fair” group (had < 5 OHS trainings)<br>- “Good” group (had 5-7 types OHS trainings)
	Breaking regulation behaviour*	Four items to examine participants’ breaking regulation behaviour	In the last 12 months, how often in your job did you drive in excess of the speed limit?	Binary (No/Yes to any of listed situations)
Lifestyle factors	Diet	National Health Survey (NHS) Module 13-Dietary behaviour	How many serves of fruit do you usually eat each day?	-Not meet the guideline<br>- Meet the guideline (had >= 5 serves of vegetables per day or<br>had >= 2 serves of fruit per day)
	Alcohol	Three AUDIT-C 3 screening questions (scored on a scale of 0-12)	How often do you have a drink containing alcohol?	-Low-risk alcohol use (score of 0-3)<br>-High-risk alcohol use score of 4 or greater)
	Smoking	Current behaviours related to smoking	How many cigarettes a day do you currently smoke?	-Not a smoker<br>- Smoker
	Physical activity	Duration of moderate or vigorous exercise in the past week	How many hours or minutes of moderate exercise did you do?	-Not meet the guideline<br>- Meet the guideline (>= 150 minutes of moderate-intensity aerobic physical activity or >= 75 minutes of vigorous-intensity aerobic physical activity)
Health risk factors	Sleep	Five items using select questions from the sleep disorders screening questionnaire (1 point was allocated for responses of a sleep duration of less than 6 hours, four nights or more per week having problems with sleep, and a “yes” response to nodding off or falling asleep while driving)	In the past month, on average how many hours of sleep do you get in a 24-hour period?	-Low-risk of poor sleep (score of 0-1)<br>-High-risk of poor sleep (score of 2 or greater)

	Fatigue	4 items drawn from previous surveys measured (No risk=Never and Less than once a month; Yes to risk=Monthly, Weekly and Daily/Almost daily)	How often do you become fatigued while driving for work?”	-Low-risk group (0-1 risk)<br>-High-risk group (2-4 risk)
	Medication use	Whether the participant took any medication to help sleep or combat fatigue	Do you currently take any medication to manage pain?	-No<br>-Yes (took any medication)
	Loneliness	One question measured by a 5-point Likert scale (0=Never, 1=Rarely, 2=Sometimes, 3=Very often, 4=Always).	During the past week, how often have you felt lonely?”	-No (never or rarely feel lonely)<br>-Yes (sometimes or always feel lonely

*Measured by 5-point Likert scale ((0=Never,1= Less than once a month, 2=Monthly, 3=Weekly, 4=Daily/Almost daily). All 5-point Likert scale responses were converted to dichotomous scales, referring to “Yes” (response of 3 or 4) and “No” (response of 0, 1 or 2).

Table S2: Univariate regression on high risk of fatigued driving

High-risk fatigue	Odds ratio	95% confidence interval	p-value
Personal factors			
**Age**			
< 35 years	Ref		
35-44 years	0.595	0.283-1.250	0.170
45-54 years	0.521	0.258-1.053	0.069
> 55 years	0.470	0.241-0.914	0.026
**Family status**			
No partner with no dependent children	Ref		
No partner with dependent children	0.275	0.099-0.757	0.012
Partnered with no dependent children	0.313	0.144-0.682	0.003
Partnered with dependent children	0.359	0.166-0.780	0.010
**Education level**			
Above high school	Ref		
High school and lower	1.165	0.732-1.856	0.519
**Financial stress**			
Low	Ref		
High	1.283	0.770-2.136	0.338
**Mental health condition**			
No	Ref		
Yes	1.507	0.869-2.615	0.144
**Physical health condition**			
No	Ref		
Yes	0.866	0.515-1.457	0.589
Occupational factors			
**Working hours**			
< 41 hours	Ref		
41-60 hours	2.419	1.189-4.920	0.015
> 60 hours	3.059	1.455-6.431	0.003
**Payment type**			
Flat rate	Ref		
Per trip/delivery	0.789	0.377-1.689	0.556
Single time pay	0.884	0.497-1.574	0.676
Kilometre rate	1.676	0.866-3.245	0.125
Other	2.346	0.921-5.977	0.074
**Shift type**			
Multiple trips between same location	Ref		
Single long trip between 2 locations	1.472	0.860-2.520	0.158
Multiple trips between 2 locations	1.185	0.668-2.101	0.562
**Employment type**			
Owner driver	Ref		
Not owner driver	1.121	0.606-2.076	0.714
**Driving experience**			
< 5 years	Ref		
5-20 years	1.370	0.655-2.884	0.406
> 20 years	0.570	0.286-1.135	0.110
**Work task**			
Low risk	Ref		
Moderate risk	2.402	1.439-4.009	0.001
High risk	4.067	0.614-3.456	<0.001
**Work time and schedule**			
Low risk	Ref		
High risk	1.907	1.205-3.019	0.006
Environmental factors			
**Work environment**			
Low-risk	Ref		
High-risk	2.732	1.632-4.575	<0.001
**Workplace violence**			

No	Ref		
Yes	0.746	1.193-2.990	0.007
Regulatory factors			
**Breaking rules regulation**			
No	Ref		
Yes	2.976	1.8667-4.746	<0.001
**Fatigue management**			
No fatigue management	Ref		
Basic/advanced fatigue management	0.981	0.620-1.552	0.935
Lifestyle factors and health risk			
**Diet**			
Not meet the guideline	Ref		
Meet the guideline	0.655	0.408-1.0523	0.080
**Exercise**			
Not meet the guideline	Ref		
Meet the guideline	0.661	0.421-1.037	0.072
**Smoking**			
Non-smokers	Ref		
Smokers	2.234	1.243-4.015	0.007
**Drinking**			
Light drinker	Ref		
Heavy drinker	0.916	0.583-1.439	0.704
**Sleep**			
Low-risk	Ref		
High-risk	10.818	3.811-30.710	<0.001
**Pain**			
No	Ref		
Yes	2.344	1.466-3.749	<0.001
**Loneliness**			
No	Ref		
Yes	3.638	2.202-6.008	<0.001
**BMI (Body mass index)**			
Under or normal weight (BMI<25)	Ref		
Overweight (BMI25-29)	0.624	0.314-1.239	0.178
Obese (BMI>29)	1.154	0.621-2.145	0.650

Table S3: Univariate regression on having used something to combat fatigue

High-risk fatigue	Odds ratio	95% confidence interval	p-value
Personal factors			
**Age**			
< 35 years	Ref		
35-44 years	0.891	0.447-1.775	0.743
45-54 years	0.352	0.178-0.713	0.004
> 55 years	0.233	0.115-0.469	<0.001
**Family status**			
No partner with no dependent children	Ref		
No partner with dependent children	1.2	0.472-3.054	0.702
Partnered with no dependent children	0.327	0.156-0.682	0.003
Partnered with dependent children	0.661	0.337-1.297	0.229
**Education level**			
Above high school	Ref		
High school and lower	0.667	0.399-1.115	0.123
**Financial stress**			
Low	Ref		
High	2.241	1.340-3.749	0.002
**Mental health condition**			
No	Ref		
Yes	1.724	0.999-2.974	0.050
**Physical health condition**			
No	Ref		
Yes	1.153	0.660-2.015	0.617
Occupational factors			
**Working hours**			
< 41 hours	Ref		
41-60 hours	0.778	0.355-1.699	0.528
> 60 hours	1.386	0.629-3.053	0.419
**Payment type**			
Flat rate	Ref		
Per trip/delivery	2.269	0.995-5.177	0.051
Single time pay	1.609	0.822-3.149	0.165
Kilometre rate	1.897	0.930-3.870	0.078
Other	2.432	1.014-5.829	0.046
**Shift type**			
Multiple trips between same location	Ref		
Single long trip between 2 locations	1.392	0.795-2.438	0.247
Multiple trips between 2 locations	1.364	0.740-2.515	0.319
**Employment type**			
Owner driver	Ref		
Not owner driver	0.943	0.489-1.818	0.862
**Driving experience**			
< 5 years	Ref		
5-20 years	0.917	0.453-1.854	0.809
> 20 years	0.345	0.170-0.702	0.003
**Work task**			
Low-risk	Ref		
Moderate-risk	2.644	1.424-4.907	0.002
High-risk	2.836	1.401-5.739	0.004
**Work time and schedule**			
Low-risk	Ref		
High-risk	1.799	1.069-3.028	0.027
Environmental factors			
**Work environment**			
Low-risk	Ref		
High-risk	5.608	2.476-12.701	<0.001
**Workplace violence**			

No	Ref		
Yes	2.926	1.784-4.797	<0.001
Regulatory factors			
**Breaking rules regulation**			
No	Ref		
Yes	2.237	1.309-3.824	0.003
**Fatigue management**			
No fatigue management	Ref		
Basic/advanced fatigue management	1.186	0.721-1.949	0.502
Lifestyle factors and health risk			
**Diet**			
Not meet the guideline	Ref		
Meet the guideline	0.954	0.569-1.599	0.858
**Exercise**			
Not meet the guideline	Ref		
Meet the guideline	0.699	0.430-1.134	0.147
**Smoking**			
Non-smokers	Ref		
Smokers	2.805	1.633-4.817	<0.001
**Drinking**			
Light drinker	Ref		
Heavy drinker	1.610	0.979-2.646	0.061
**Sleep**			
Low-risk	Ref		
High-risk	2.691	1.499-4.830	0.001
**Pain**			
No	Ref		
Yes	2.290	1.310-4.005	0.004
**Loneliness**			
No	Ref		
Yes	2.122	1.306-3.447	0.002
**BMI (Body mass index)**			
Under or normal weight (BMI<25)	Ref		
Overweight (BMI25-29)	0.693	0.339-1.416	0.314
Obese (BMI>29)	0.605	0.322-1.137	0.119
